# Machine-learning based Computed Tomography Radiomics Nomogram for Predicting Perineural Invasion in Gastric Cancer

**DOI:** 10.2174/0115734056323323250102073559

**Published:** 2025-01-13

**Authors:** Pei Huang, Sheng Li, Zhikang Deng, Fangfang Hu, Di Jin, Situ Xiong, Bing Fan

**Affiliations:** 1 From Medical College of Nanchang University, Nanchang University, Nanchang, China; 2 Department of Radiology, Jiangxi Provincial People’s Hospital, The First Affiliated Hospital of Nanchang Medical College, Nanchang, China; 3 The First Affiliated Hospital of Nanchang University, Nanchang, China

**Keywords:** Gastric cancer, Perineural invasion, Machine learning, Nomogram, Radiomics, Contrast-enhanced computed tomography

## Abstract

**Objective:**

The aim of this study was to develop and validate predictive models for perineural invasion (PNI) in gastric cancer (GC) using clinical factors and radiomics features derived from contrast-enhanced computed tomography (CE-CT) scans and to compare the performance of these models.

**Methods:**

This study included 205 GC patients, who were randomly divided into a training set (n=143) and a validation set (n=62) in a 7:3 ratio. Optimal radiomics features were selected using the least absolute shrinkage and selection operator (LASSO) algorithm. A radiomics model was constructed utilizing the optimal among five machine learning filters, and a radiomics score (rad-score) was computed for each participant. A clinical model was built based on clinical factors identified through multivariate logistic regression. Independent clinical factors were combined with the rad-score to create a combined radiomics nomogram. The discrimination ability of the models was evaluated by receiver operating characteristic (ROC) curves and the DeLong test.

**Results:**

Independent predictive factors of the clinical model included tumor T stage, N stage, and tumor differentiation, with AUC values of 0.777 and 0.809 in the training and validation sets. The radiomics model was constructed using the support vector machine (SVM) classifier with the best AUC (0.875 in the training set and 0.826 in the validation set). The combined radiomics nomogram, which combines independent clinical predictors and the rad-score, demonstrated better predictive performance (AUC=0.889 in the training set; AUC=0.885 in the validation set).

**Conclusion:**

The nomogram integrating independent clinical predictors and CE-CT radiomics was constructed to predict PNI in GC. This model demonstrated favorable performance and could potentially assist in prognosis evaluation and clinical decision-making for GC patients.

## 1 INTRODUCTION

Gastric cancer (GC) is one of the most prevalent malignant tumors of the digestive system, ranking as the fifth and third malignancy in incidence and mortality rates worldwide, respectively [1]. According to the latest reported statistics, GC resulted in approximately 769,000 tumor-related deaths in 2020 [1]. Surgical removal represents the preferred treatment approach for individuals diagnosed with gastric cancer [2]. Combining neoadjuvant chemotherapy can effectively reduce locoregional tumor dissemination and the potential for disease relapse [3, 4]. Nonetheless, the prognosis for patients with gastric cancer continues to be discouraging [5].

Accumulating research highlighted the significance of perineural invasion (PNI), which involves tumor cell clusters in the nerve fiber bundles and perineurium and plays an essential role in guiding preoperative or postoperative adjuvant therapy and predicting prognosis for GC [6-9]. Furthermore, several scholars suggested incorporating PNI into the TNM staging system to more proficiently assess the risk of recurrence among GC patients [6, 10]. Nonetheless, postoperative pathological examination is the only means to confirm PNI status. Therefore, evaluating the PNI status of GC patients is significant feasibly and accurately before surgery for assessing the risk of recurrence, predicting prognosis, and guiding personalized treatment.

Radiomics is an emerging field in radiology that involves the high-throughput extraction of quantitative features from medical images to transform them into analyzable data. Subsequent analysis of this data enhances diagnostic, prognosis, and prediction accuracy in clinical practice [11, 12]. Recently, radiomics has been applied to predict the PNI status in colorectal cancer, prostate cancer, cholangiocarcinoma, *etc* [13-17], but its utilization in gastric cancer remains limited [18, 19]. Consequently, this research aims to construct and validate a radiomics-based nomogram that amalgamates both radiomic signatures derived from contrast-enhanced computed tomography(CE-CT)images and pertinent clinical indicators to predict PNI status in gastric cancer (GC) with a high degree of precision.

## 2 MATERIALS AND METHODS

### 2.1 Patients

The ethics committee at our clinical center approved the research and waived the requirement for written informed consent from participants. This retrospective study consecutively collected data from patients who underwent radical gastrectomy and D2 lymph node dissection from January 2018 to October 2022 at our clinical center.

The exclusion criteria were: (1) received any anti-tumor treatment prior to surgery; (2) lesions smaller than 10mm could not be delineated; (3) there exist imaging artifacts that may impact the analysis of the image; (4) the abdominal CE-CT examination was performed over 14 days before the surgery; (5) residual gastric cancer; (6) inadequate gastric distension; and (7) clinical data were incomplete.

From a medical database, the clinical and pathological data were extracted, which included gender, age, tumor size, tumor location, T stage, N stage, histological grade, and detection results of cancer antigen (CA) 199, carcinoembryonic antigen (CEA), and alpha-fetoprotein (AFP).

**Fig. (1):**
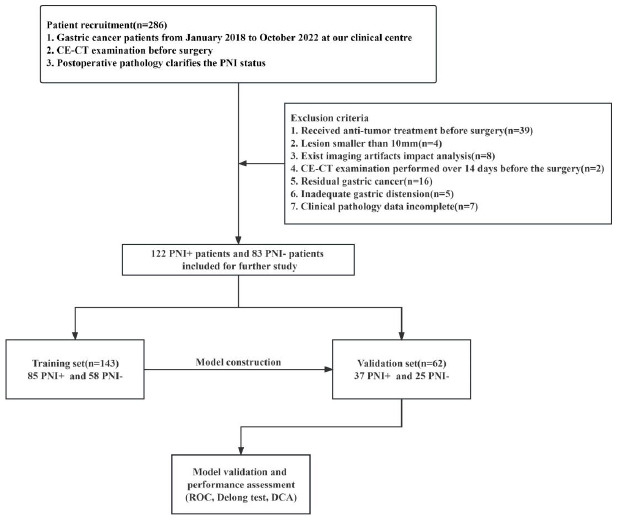
Patient recruitment and research workflow.

Eventually, this study included 205 GC patients (122 PNI positive and 83 PNI negative; 144 males and 61 females; median age 63.00 ± 10.39 years). These patients were randomly split into training or a validation cohort, following a 7:3 distribution (Fig. **1**).

### 2.2 Clinical Model Construction

To identify the independent clinical predictive factors associated with PNI status in the training set, we utilized univariable and multivariable logistic regression to analyze the clinical factors. Subsequently, these independent variables were utilized to develop the clinical model using the logistic regression algorithm.

### 2.3 CE-CT Image Acquisition

A Siemens SOMATOM Definition CT scanner was used to capture abdominal contrast-enhanced CT scan images. Prior to the examination, the patient was instructed to drink 500-1000ml of water to distend the stomach. The scan parameters were automatically adjusted as follows: Tube voltage of 120 kV, tube current of 150 mAs, slice thickness of 1.25 mm, reconstruction interval of 1 mm, and layer spacing of 1 mm. Contrast-enhanced CT images in the venous phase were acquired 60 seconds after the contrast agent was injected through the antecubital vein.

### 2.4 ROI Segmentation

**Fig. (2):**
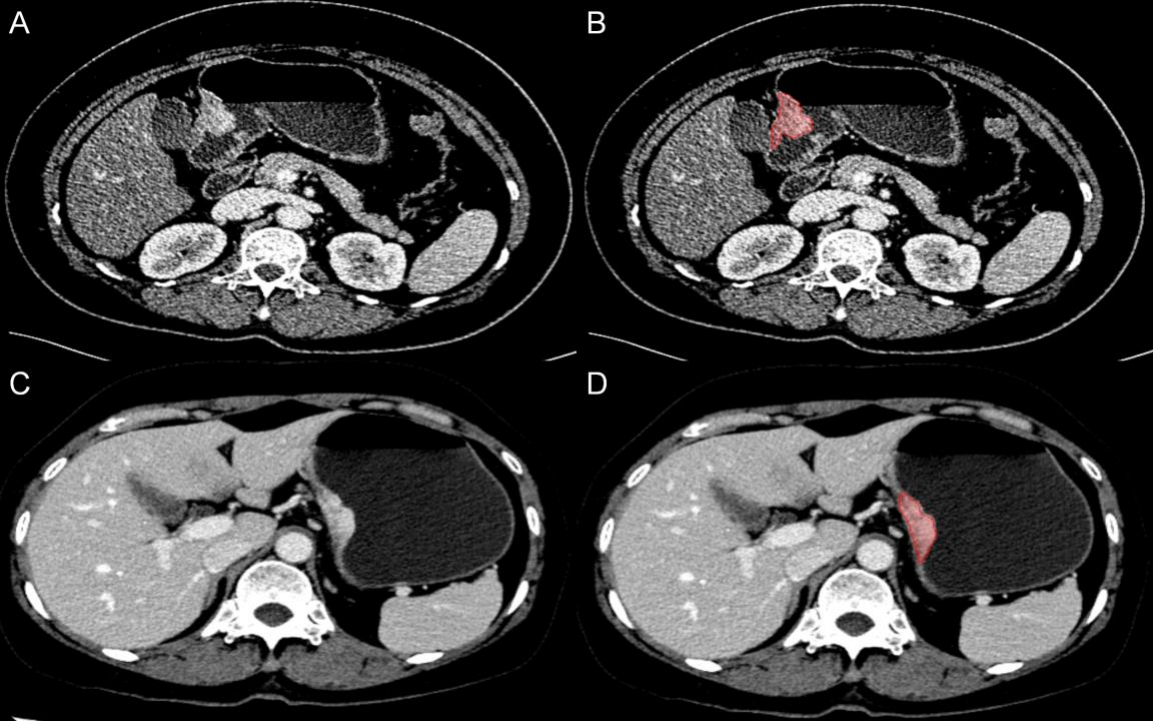
The tumor outline, red area signifies the region of interest (ROI) of tumor. Case 1: gastric cancer patient with PNI negative, female, 51 years old (**A**). Manual delineate the ROI of the lesion (**B**). Case 2: gastric cancer patient with PNI positive, male, 53 years old (**C**). Manual delineate the ROI of the lesion (**D**).

On the imported venous phase CE-CT images, the area of gastric cancer was delineated manually using the DARWIN scientific research platform (Beijing Yizhun Intelligent Technology Co., LTD., China, https://arxiv.org/abs/2009.00908). In a meticulous procedure, a radiologist with 6 years of expertise in gastroenterology delineated the tumor's region of interest (ROI) in a stepwise, layer-by-layer manner. Following this, a highly experienced senior radiologist with over 15 years of gastroenterology specialization reassessed and refined the identified ROI. The contour of the lesion was meticulously delineated by the senior radiologist when a discrepancy arose in the segmentation boundary (Fig. **2**). Neither of the radiologists had prior awareness of the pathological outcomes of the lesions.

### 2.5 Extraction and Selection of Radiomic Features

For each lesion, a sum of 1781 radiomics features was extracted using the Pyradiomics package (version 3.1.0) in Python, which was incorporated into the previously mentioned platform. The radiomics features comprised first-order, shape, and texture features extracted from the original image. Additionally, it included the first-order and texture features generated by applying 8 types of filters processed images: logarithm, gradient, square, square root, exponential, wavelet, local binary pattern (LBP), and Laplacian of Gaussian (LoG). Texture features include gray-level co-occurrence matrix (GLCM), gray-level dependence matrix (GLDM), gray-level run-length matrix (GLRLM), neighboring gray-tone difference matrix (NGTDM), and gray-level size zone matrix (GLSZM) features.

**Fig. (3):**
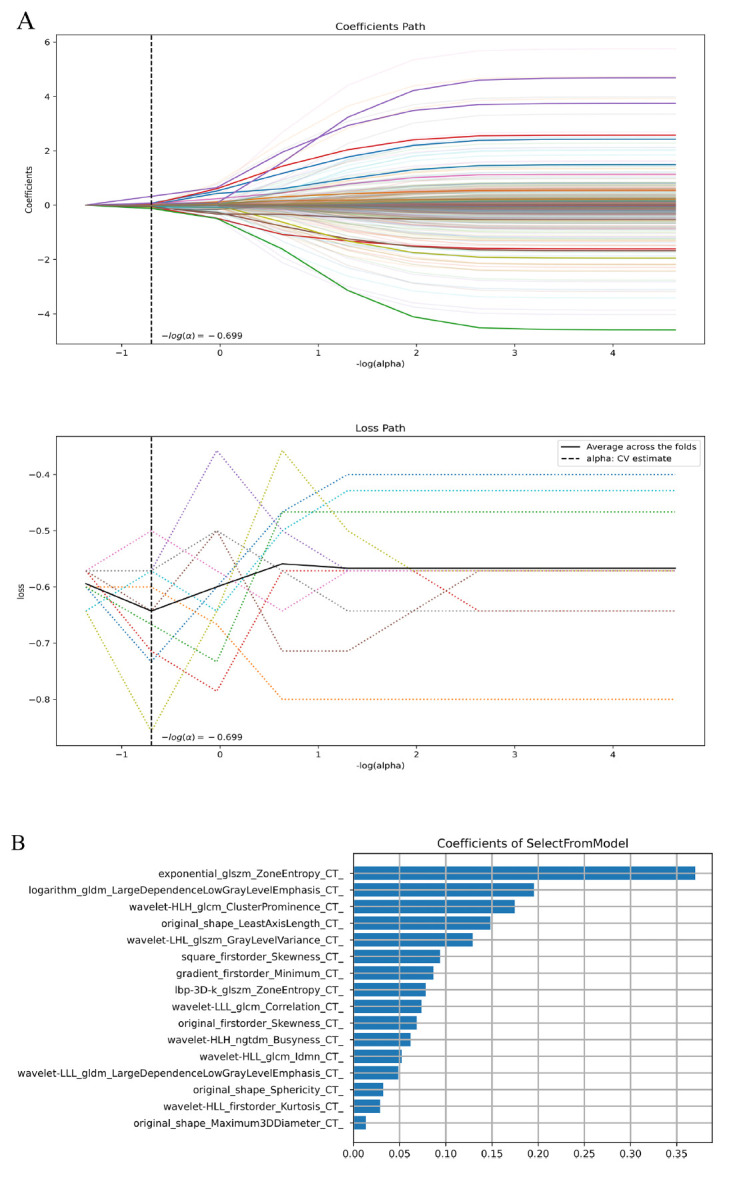
The finalized radiomic features (**B**) derived from the LASSO algorithm (**A**) for predicting PNI in GC.

Prior to the selection of the extracted features, the dataset underwent a process of Z-score standardization to ensure uniformity. Then, further feature selection was performed using a two-step method. Initially, the premier feature filter, ascertained by the F-value corresponding to the variance of samples within the training dataset, was employed to evaluate the linear association of each feature with the categorical labels, aiming to pinpoint the most salient features. From an initial pool of 1781 features, a total of 576 significant features were identified and chosen. In the subsequent phase, the least absolute shrinkage and selection operator (LASSO) regression was applied to select the optimal features that exhibited non-zero coefficients (Fig. **3A**). In the final analysis, the LASSO regression was executed with an optimally tuned regularization parameter α set at -0.699, successfully differentiating a set of 16 features with non-zero coefficients that are pertinent to the PNI status in GC, as illustrated in Fig. (**3B**).

### 2.6 Development of Radiomics and Combined Models

Based on the selected optimal radiomic features, five distinct machine learning algorithms were developed, namely k-nearest neighbor (KNN), logistic regression (LR), extreme gradient boosting (XGBoost), support vector machine (SVM), and random forest (RF). The prediction performance of the five models received a further assessment *via* the validation set. To avoid model overfitting, we implemented the ten-fold cross-validation approach in developing the models. The efficacy of the five predictive models was assessed by measuring the area under the curve (AUC) of the receiver operating characteristic (ROC) curve. In addition, the models' sensitivity, specificity, and overall accuracy were also calculated. The model with the highest performance metrics was selected to develop the radiomics model. Among the training and validation sets, the radiomics score (Rad-score) was computed. To establish the combined model (combined radiomics nomogram), the independent clinical predictive factors and rad-score were integrated *via* logistic regression.

### 2.7 Model Performance Assessment

The discrimination performance of the three models (clinical model, radiomics model, and combined nomogram) to predict PNI status was evaluated with the AUC values of the ROC curves. DeLong tests were used to compare the model's performance. Calibration plots were obtained to evaluate the agreement between predicted and pathological status. To assess clinical utility, we employed decision curve analysis (DCA) to determine the net benefit of the models across various reasonable threshold probabilities.

### 2.8 Statistical Analyses

Statistical evaluations were conducted employing SPSS software (version 24.0) and R software (version 4.1.0). To evaluate normally distributed continuous variables, reported as mean and standard deviation, we applied the t-test. Non-normally distributed data, described as median and interquartile range, were compared utilizing the Wilcoxon rank-sum test. In the case of categorical variables, the chi-square test was employed, and the results were reported as counts and percentages. A *p*-value of less than 0.05 was considered to be statistically significant. The Spearman correlation coefficient was calculated to assess the redundancy between features included in the optimal model.

## 3 RESULTS

### 3.1 Patient Characteristics

Among the 205 patients with gastric cancer (GC), 143 individuals (including 85 with PNI positive and 58 with PNI negative) were allocated to the training set. In contrast, 62 others (37 with PNI positive and 25 with PNI negative) were assigned to the validation set randomly. A comprehensive list of all clinicopathological features is detailed in Table **1**. No significant disparities (*p*>0.05) were observed between the training and validation sets. In the training set, the PNI-positive group had a higher female proportion (38.8% *vs* 19.0%, *p*=0.012) and a significant correlation with tumor location (*p*=0.03). PNI positivity was also associated with more advanced T stage (87.1% *vs* 58.6%, *p*<0.001) and higher prevalence of lymph node metastasis (87.1% *vs* 48.3%, *p*<0.001). Additionally, the PNI-positive group exhibited poorer tumor differentiation (75.3% *vs* 50.0%, *p*=0.002). No significant deviations were noted in the other clinicopathologic characteristics between these two groups (*p*>0.05).

### 3.2 Development of the Three Predictive Models for PNI Status

After performing multivariable logistic regression analysis (Table **2**), T stage (OR, 3.48; 95%Cl, 1.33-9.08; *p*=0.011), N stage (OR, 4.92; 95%Cl, 2.01-12.04; *p*<0.001) and tumor differentiation (OR, 0.34; 95% Cl, 0.15-0.78; *p*=0.011) was determined as independent predictors of PNI status and the clinical model was developed. The predictive abilities of the five machine learning classifiers are presented in Table S1. Comparing the AUC values of the five classifiers revealed that the SVM classifier had the best predictive performance in the validation set (Fig. **S1**). The AUC, sensitivity, specificity and accuracy were 0.826, 0.811, 0.800, and 0.806, respectively. Therefore, the radiomics model was developed utilizing the SVM classifier. As depicted in Fig. (**S2**), a statistically significant difference in the rad-score was observed between patients with PNI-positive and PNI-negative status. This disparity was consistent across both the training and validation sets (*p*<0.001), indicating the association between radiomic features and the PNI status in GC. The formula of the rad-score is provided in Appendix **S1**.

**Fig. (4):**
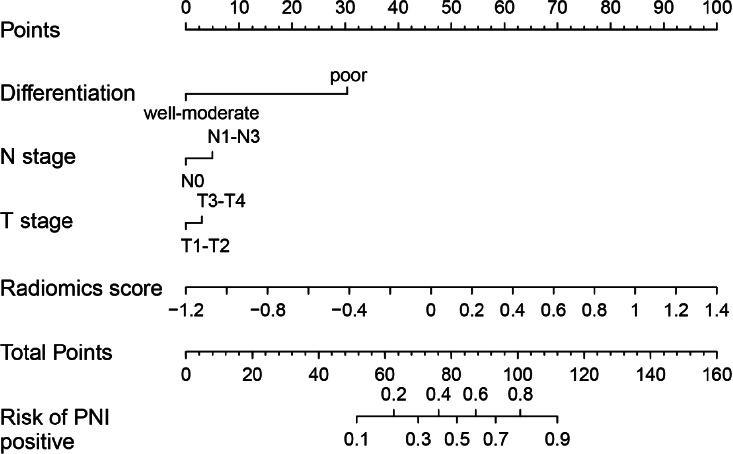
Combined model (combined radiomics nomogram) for assessing the risk of PNI in individuals with GC.

To create a more precise and clinically applicable model, the combined model was constructed using the logistic regression algorithm. This model integrated rad-score with independent clinical predictors. The variables incorporated in the combined model were presented in a nomogram to individualize the prediction of PNI status in GC (Fig. **4**). As shown in Appendix **S2**, the Spearman correlation coefficients for T stage, N stage, tumor differentiation, and rad-score range from -0.144 to 0.485, indicating that the redundancy among these variables is relatively small.

### 3.3 Assessment of the Effectiveness of the Diverse Models

**Fig. (5):**
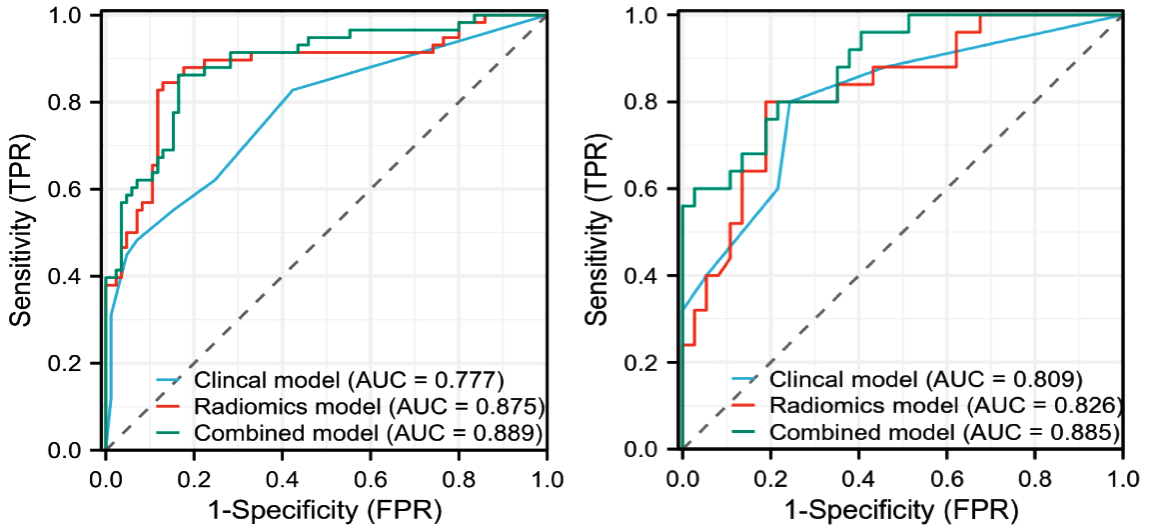
AUC values for clinical, radiomics and combined models in the training set (left) and validation set (right) to discriminate PNI status.

**Fig. (6):**
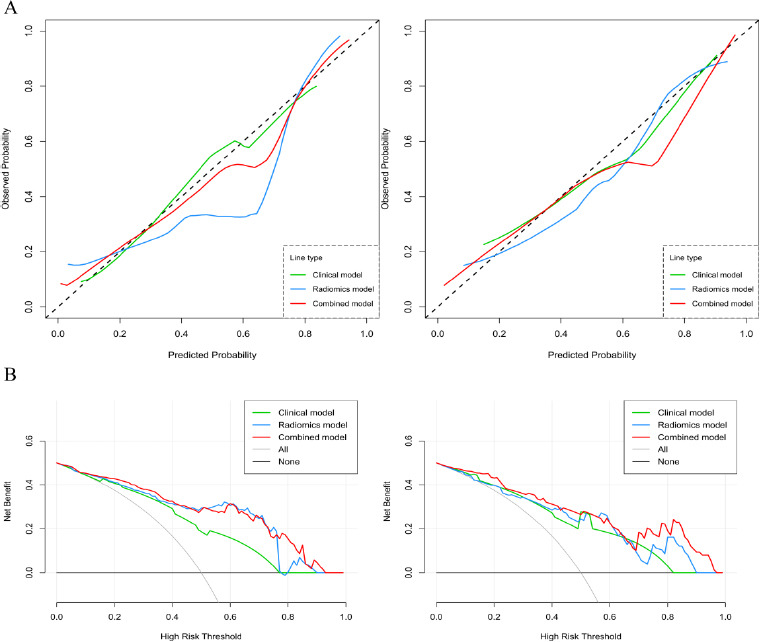
Calibration curve of clinical, radiomics and combined PNI-prediction models in the training set (**A**, left) and validation set (**A**, right). DCA of clinical, radiomics and combined PNI-prediction models in the training set (**B**, left) and validation set (**B**, right).

The AUC values for the clinical model, radiomics model, and combined model were 0.777, 0.875, 0.889 in the training set and 0.809, 0.826, 0.885 in the validation set (Table **3** and Fig. **5**). As shown in Table **S2**, the Delong test indicated that in the training set, the AUC values of the radiomics and combined models exceeded the clinical model significantly (*p*=0.026 and *p*<0.001, respectively). However, no significant statistical differences were observed between the radiomics model and the combined model in either the training or validation sets (*p* > 0.05). It can be seen from the calibration plots a strong agreement between the PNI status predicted by the model and the actual PNI status across all datasets and models (Fig. **6A**). As demonstrated by Fig. (**6B**)'s DCA results, the combined model outperforms the other models in effectively identifying between PNI-positive and PNI-negative gastric cancer patients, proving its superiority across varied reasonable threshold probabilities.

## 4 DISCUSSION

Perineural invasion (PNI) is considered one of the significant factors in evaluating the prognosis and treatment response in gastric cancer. The results of the current investigation demonstrated that the clinical model exhibited AUC values of 0.777 in the training set and 0.809 in the validation set. This suggests that the model is well-suited for predicting PNI in patients with GC. Additionally, when radiomics features were incorporated into the clinical model, it resulted in a notable improvement in its predictive efficacy, with AUC values of 0.889 in the training set and 0.885 in the validation set, respectively. The notable enhancement in predictive precision and overall net benefit of the model highlights the potential of integrating radiomics into clinical models to improve accuracy and performance.

To accurately detect high-risk recurrence of GC and thereby enhance individualized treatment strategies accordingly, previous studies have employed various methods to predict PNI preoperatively. Several researchers utilized clinical characteristics to predict PNI in GC and demonstrated good predictive performance. Liu *et al*. [20]. constructed a nomogram to predict PNI in gastric cancer patients using Carcinoembryonic Antigen(CEA), tumor length, T stage, lymph node metastasis, and Lauren classification. However, only a predictive nomogram containing clinical features was constructed without exploring the potential improvement by incorporating additional histological information into the models. Li *et al*. [21] developed enhanced spectral CT-based parameters to predict the PNI status in GC patients, which exhibited the highest AUC of 0.782 in the validation cohort. Although the nomogram is somewhat innovative in terms of methodology, the relatively high cost of spectral CT equipment may limit its widespread adoption and application in most medical institutions. In contrast, radiomics can comprehensively evaluate the heterogeneity of tumors in a non-invasive, economical, and easily available way. Zheng *et al*. [18] established a combined radiomics model that utilized CE-CT for estimating the PNI status in patients with gastric cancer. This model demonstrated AUC values of 0.90 in the training set and 0.82 in the testing set. Nevertheless, only LR and SVM machine learning classifiers in this study were employed to construct the radiomics model and did not investigate the potential of other alternative classifiers. Therefore, this research evaluated five machine learning classifiers to determine the optimal one. Then, this chosen classifier was incorporated with relevant clinical characteristics to construct a comprehensive CE-CT-based radiomics nomogram, aiming to improve the prediction of PNI in GC.

This research developed a clinical model for estimating PNI in GC, which incorporated T stage (OR, 3.48; 95% CI, 1.33-9.08; *P* = 0.011), N stage (OR, 4.92; 95% CI, 2.01-12.04; *p* < 0.001), and tumor differentiation (OR, 0.34; 95% CI, 0.15-0.78; *P* = 0.011). No significant differences were found in the other clinical characteristics. Several studies have highlighted the significant association between the T stage, N stage, and the PNI status. Both Liu *et al*. and Li *et al*. have recognized T staging and lymph node metastasis of gastric cancer as independent predictive factors for PNI [20, 22]. The research by Luo *et al*. examined the relationship between PNI and overall survival following gastric resection in 1632 gastric cancer patients. It revealed that the proportion of PNI-positive individuals in the T1-T2 stage was 16.5%, while it was 43.4% in the T3-T4 stage [23]. Moreover, increasing evidence has pointed out that lower differentiation correlates with a higher PNI positivity rate [8, 24]. The above confirms the reliability and validity of the independent predictors utilized in constructing the clinical model for PNI of GC.

This research established a radiomics model using the LASSO algorithm composed of 16 chosen features (9 texture elements, 4 first-order elements, and 3 shape elements). In differentiating between PNI-positive and PNI-negative GC, the gray-level size zone matrix (GLSZM) carried the most significant weight score. GLSZM determines the number of adjacent voxels with identical gray intensity by measuring the gray region in the digital image. This feature characterizes the unevenness within the tumor, which is a strong clue of high heterogeneity and reliable markers of the aggressive nature of tumors [25]. Its efficacy has been verified by many previous published studies [26, 27]. Moreover, 7 wavelet features, which were derived through the process of wavelet decomposition of the original image, were selected in our research. The wavelet features can detect information about texture heterogeneity and demonstrate a tendency associated with tumor heterogeneity [28], which has been effectively employed in computer-assisted diagnostic systems for a range of malignant tumors [29, 30]. Previous studies stated that GC with a higher degree of microenvironmental heterogeneity presents a worse prognosis [31, 32]. As mentioned above, the prognosis of GC patients is affected by the PNI status [8, 9, 24]. This clarifies why these radiomics features, which reflect the information on tumor heterogeneity, are capable of predicting the PNI status.

Our study has some limitations. First, it is retrospective, which may introduce potential biases. Furthermore, the sample is collected from one medical facility involving a relatively small number of patients. Consequently, a multi-center, prospective, and large-scale investigation is essential to reduce regional disparities and validate the models. Finally, the CE-CT images were exclusively acquired using the Siemens SOMATOM Definition CT scanner. Further verification is essential to assess the reproducibility and performance of the model on images acquired from other CT scanners.

## CONCLUSION

The machine learning-based radiomics model developed in this study demonstrated excellent predictive accuracy and reliability, effectively assessing the correlation between imaging and postoperative pathological PNI status in a non-invasive manner. The combined radiomics nomogram, which integrates radiomics features with clinical characteristics, further improves the accuracy of predicting PNI status in GC patients. This approach has the potential to aid in prognosis evaluation and guide clinical decision-making for GC patients.

## Figures and Tables

**Table 1:** Patient clinicopathological characteristics in the training and validation sets. Highlighted numbers indicated statistically difference (*p*<0.05).

-	**Training Set (n=143)**			***P-value***	**Validation Set (n=62)**	P*-value
	**Total**	**PNI positive**	**PNI negative**			
Age,years,mean(SD)	63.06±10.17	62.11±11.23	64.47±8.27	0.151	62.87±10.97	0.904
Gender,n(%)				**0.012**		0.630
male	99(69.23)	52(61.18)	47(81.03)		45(72.58)	
female	44(30.77)	33(38.82)	11(18.97)		17(27.42)	
Size,n(%)				0.196		0.555
≤4cm	72(50.35)	39(45.88)	33(56.90)		34(54.84)	
>4cm	71(49.65)	46(54.12)	25(43.10)		28(45.16)	
Location,n(%)				**0.030**		0.328
upper	11(7.69)	8(9.41)	3(5.17)		8(12.90)	
middle	42(29.37)	31(36.47)	11(18.97)		21(33.87)	
lower	90(62.94)	46(54.12)	44(75.86)		33(53.23)	
Differentiation,n(%)				**0.002**		0.548
well-moderate	50(34.97)	21(24.71)	29(50.00)		19(30.65)	
poor	93(65.03)	64(75.29)	29(50.00)		43(69.35)	
T stage,n(%)				**<0.001**		0.77
T1-T2	35(24.48)	11(12.94)	24(41.38)		14(22.58)	
T3-T4	108(75.52)	74(87.06)	34(58.62)		48(77.42)	
N stage,n(%)				**<0.001**		0.232
N0	41(28.67)	11(12.94)	30(51.72)		23(37.10)	
N1-N3	102(71.33)	74(87.06)	28(48.28)		39(62.90)	
AFP,n(%)				0.102		0.410
≤5.0 mg/ml	126(88.11)	78(91.76)	48(82.76)		52(83.87)	
>5.0 mg/ml	17(11.89)	7(8.24)	10(17.24)		10(16.13)	
CEA,n(%)				0.889		0.855
≤5.0 mg/ml	102(71.33)	61(71.76)	41(70.69)		45(72.58)	
>5.0 mg/ml	41(28.67)	24(28.24)	17(29.31)		17(27.42)	
CA199				0.337		0.262
≤27.0 mg/ml	118(82.52)	68(80.00)	50(86.21)		55(88.71)	
>27.0 mg/ml	25(17.48)	17(20.00)	8(13.79)		7(11.29)	
Rad-score, median (IQR)	0.90 (0.13, 1.00)	1.00 (0.90, 1.01)	0.04 (-1.00, 0.58)	**<0.001**	0.56 (-0.14, 0.83)	0.081

**Table 2:** Investigation of the relationship between various characteristics and perineural invasion through logistic regression analysis in the training set.

-	**Univariate Analysis**		**Multivariate Analysis**	
	**OR(95%** **Cl)**	***P-value***	**OR(95%** **Cl)**	***P-value***
Age	0.98(0.94-1.01)	0.174		
Gender	2.71(1.23-5.96)	**0.013**	2.28(0.93-5.60)	0.073
Size	1.56(0.79-3.05)	0.197		
Location	0.49(0.27-0.89)	**0.019**	0.62(0.31-1.21)	0.161
Differentiation	0.33(0.16-0.67)	**0.002**	0.34(0.15-0.78)	**0.011**
T stage	4.75(2.09-10.79)	**<0.001**	3.48(1.33-9.08)	**0.011**
N stage	7.21(3.19-16.31)	**<0.001**	4.92(2.01-12.04)	**<0.001**
AFP	0.43(0.15-1.21)	0.109		
CEA	0.95(0.45-1.98)	0.889		
CA199	1.56(0.63-3.91)	0.340		

**Table 3:** Predictive performance of clinical, radiomics, and combined models in training and validation sets.

-	**Training set**				**Validation set**			
	**SEN**	**SPE**	**ACC**	**AUC**	**SEN**	**SPE**	**ACC**	**AUC**
Clinical	0.659	0.759	0.699	0.777	0.541	0.880	0.677	0.809
Radiomics	0.871	0.828	0.853	0.875	0.811	0.800	0.806	0.826
Combined	0.824	0.862	0.840	0.889	0.784	0.880	0.822	0.885

**Abbreviations: **SEN, sensitivity; SPE, specificity; ACC, accuracy; AUC, area under the curve.

## Data Availability

The data and supportive information are available within the article.

## LIST OF ABBREVIATIONS

PNI: Perineural Invasion
GC: Gastric Cancer
CE-CT: Contrast-enhanced Computed Tomography
AUC: Area Under The Curve

## STANDARDS OF REPORTING

STROBE guidelines were followed.
