# Supplementary material for: Machine-learning based Computed Tomography Radiomics Nomogram for Predicting Perineural Invasion in Gastric Cancer
Source: Curr Med Imaging. 2025 Jan 13;21:e15734056323323. doi: 10.2174/0115734056323323250102073559 (PMC12933236; doi:10.2174/0115734056323323250102073559)
Supplement: Supplementary file 1 [file CMIM-21-E15734056323323_SD1.pdf]

1573-4056/25 *Send Orders for Reprints to reprints@benthamscience.net*

BENTHAM SCIENCE

CrossMark

Current Medical Imaging

Content list available at: https://benthamscience.com/journals/cmir

Supplementary Material

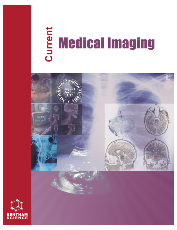

Machine-Learning based Computed Tomography Radiomics Nomogram for Predicting Perineural Invasion in Gastric Cancer

Pei Huang[1,2,#], Sheng Li[1,3,#], Zhikang Deng[1,2,#], Fangfang Hu[2], Di Jin[1,2], Situ Xiong[1,3,*] and Bing Fan[2,*]

[1]*From Medical College of Nanchang University, Nanchang University, Nanchang, China*
[2]*Department of Radiology, Jiangxi Provincial People's Hospital, The First Affiliated Hospital of Nanchang Medical College, Nanchang, China*
[3]*The First Affiliated Hospital of Nanchang University, Nanchang, China*

Appendix S1

The calculation formula for the radscore is as follows:

RadScore=+0.417

+0.374*exponential_glszm_ZoneEntropy -0.195*logarithm_gldm_LargeDependenceLowGrayLevelEmphasis

-0.175*wavelet-HLH_glcm_ClusterProminence +0.149*original_shape_LeastAxisLength

-0.131*wavelet-LHL_glszm_GrayLevelVariance

-0.093*square_firstorder_Skewness

-0.086*gradient_firstorder_Minimum

+0.078*lbp-3D-k_glszm_ZoneEntropy

+0.073*wavelet-LLL_glcm_Correlation

-0.068*original_firstorder_Skewness

+0.062*wavelet-HLH_ngtdm_Busyness

-0.051*wavelet-HLL_glcm_Idmn

-0.049*wavelet-LLL_gldm_LargeDependenceLowGrayLevelEmphasis

-0.033*original_shape_Sphericity

+0.029*wavelet-HLL_firstorder_Kurtosis

+0.013*original_shape_Maximum3DDiameter

Appendix S2

The Spearman rank correlation coefficient between T stage and N stage is 0.289. For T stage and Differentiation, the coefficient is -0.012. The correlation between T stage and rad-score is 0.485. Between N stage and Differentiation, the coefficient is -0.144, and between N stage and rad-score, it is 0.394. The correlation between Differentiation and rad-score is -0.061.

Table S1. Predictive performance comparison of five machine learning classifiers.

	Training set				Validation set			
	SEN	SPE	ACC	AUC	SEN	SPE	ACC	AUC
SVM	0.871	0.828	0.853	0.875	0.811	0.800	0.806	0.826
XGBOOST	1	0.983	0.993	1	0.622	0.760	0.677	0.719
KNN	0.906	0.603	0.783	0.838	0.703	0.880	0.774	0.776
RF	1	1	1	1	0.730	0.760	0.742	0.806
LR	0.729	0.724	0.727	0.793	0.757	0.760	0.758	0.799

Abbreviations: SVM, support vector machine; XGBOOST, eXtreme Gradient Boosting; KNN, k-Nearest Neighbor; RF, random forest; LR, logistic regression; SEN, sensitivity; SPE, specificity; ACC, accuracy; AUC, area under the curve.

DOI: 10.2174/0115734056323323250102073559, 2025, *21*, e15734056323323

Table S2. Results of the DeLong test for clinical, radiomics, and combined models.

Group	Model 1	Model 2	*P-value*
Training			
	Clinical	Radiomics	**0.026**
	Clinical	Combined	**<0.001**
	Radiomics	Combined	0.511
Validation			
	Clinical	Radiomics	0.814
	Clinical	Combined	0.103
	Radiomics	Combined	0.064

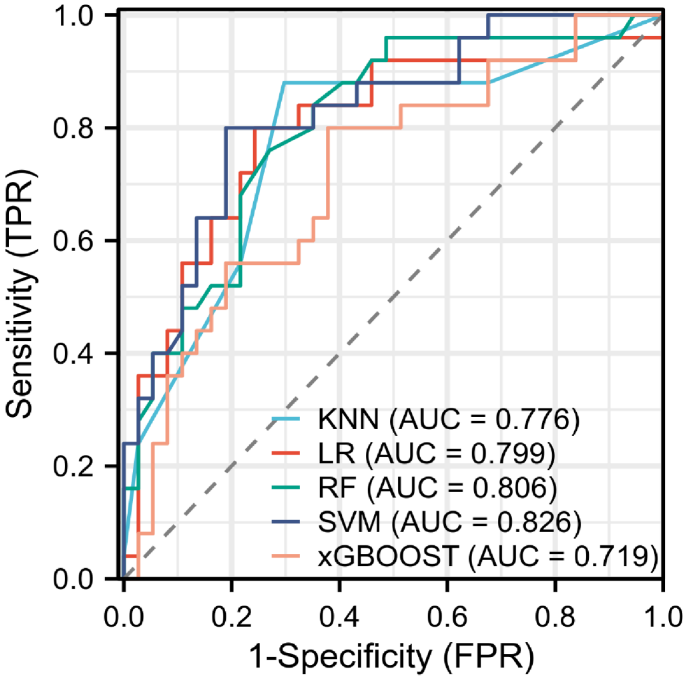

Fig. (S1). ROC curves analysis of the five machine learning classifiers within the validation set.

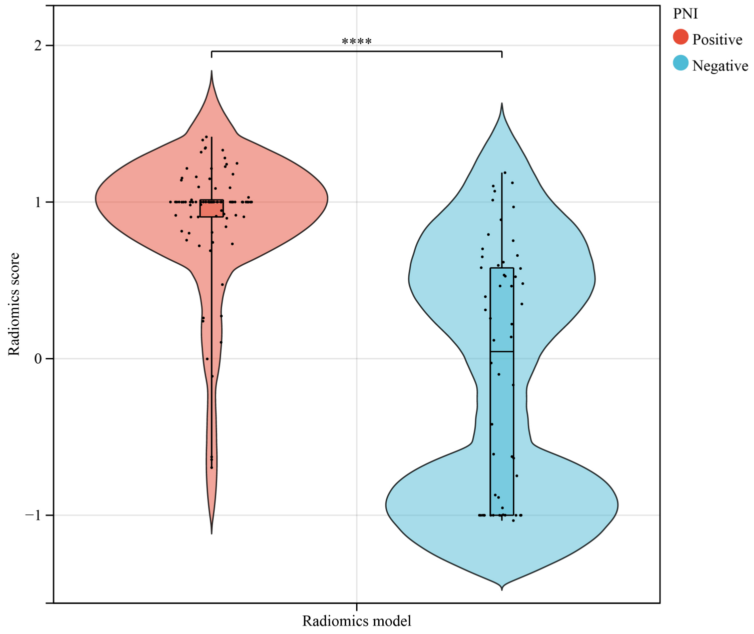

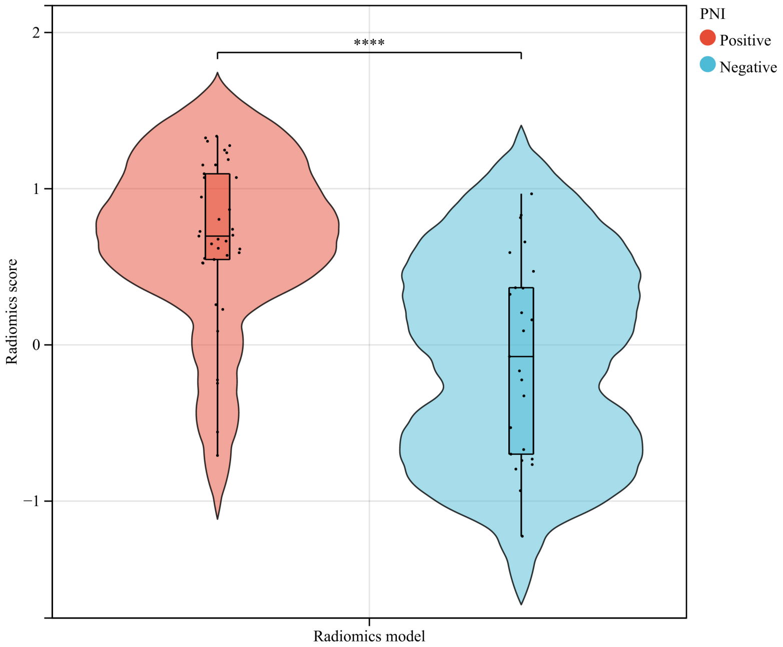

Fig. (S2). Comparison of rad-score between PNI-positive and PNI-negative gastric cancer in the training (above) and validation (below) sets. ****, *P*<0.001.

© 2025 The Author(s). Published by Bentham Science Publisher.

This is an open access article distributed under the terms of the Creative Commons Attribution 4.0 International Public License (CC-BY 4.0), a copy of which is available at: https://creativecommons.org/licenses/by/4.0/legalcode. This license permits unrestricted use, distribution, and reproduction in any medium, provided the original author and source are credited.
